# Global Phylogenomic Analysis of Nonencapsulated *Streptococcus pneumoniae* Reveals a Deep-Branching Classic Lineage That Is Distinct from Multiple Sporadic Lineages

**DOI:** 10.1093/gbe/evu263

**Published:** 2014-12-24

**Authors:** Markus Hilty, Daniel Wüthrich, Susannah J. Salter, Hansjürg Engel, Samuel Campbell, Raquel Sá-Leão, Hermínia de Lencastre, Peter Hermans, Ewa Sadowy, Paul Turner, Claire Chewapreecha, Mathew Diggle, Gerd Pluschke, Lesley McGee, Özgen Köseoğlu Eser, Donald E. Low, Heidi Smith-Vaughan, Andrea Endimiani, Marianne Küffer, Mélanie Dupasquier, Emmanuel Beaudoing, Johann Weber, Rémy Bruggmann, William P. Hanage, Julian Parkhill, Lucy J. Hathaway, Kathrin Mühlemann, Stephen D. Bentley

**Affiliations:** ^1^Institute for Infectious Diseases, University of Bern, Switzerland; ^2^Department of Infectious Diseases, Inselspital, Bern University Hospital and University of Bern, Switzerland; ^3^Interfaculty Bioinformatics Unit, University of Bern, Switzerland; ^4^Swiss Institute of Bioinformatics, Lausanne, Switzerland; ^5^Wellcome Trust Sanger Institute, Hinxton, United Kingdom; ^6^Instituto de Tecnologia Química e Biológica, University of Lisbon, Portugal; ^7^Laboratory of Microbiology and Infectious Diseases, The Rockefeller University; ^8^Laboratory of Pediatric Infectious Diseases, Radboud University Medical Centre, Nijmegen, The Netherlands; ^9^National Medicines Institute, Warsaw, Poland; ^10^Shoklo Malaria Research Unit, Mahidol-Oxford Tropical Medicine Research Unit, Faculty of Tropical Medicine, Mahidol University, Mae Sot, Thailand; ^11^Centre for Tropical Medicine, Nuffield Department of Medicine, University of Oxford, United Kingdom; ^12^Clinical Microbiology Department, Queens Medical Centre, Nottingham, United Kingdom; ^13^Swiss Tropical and Public Health Institute, University of Basel, Switzerland; ^14^Respiratory Diseases Branch, Centers for Disease Control and Prevention, Georgia, Atlanta; ^15^Department of Microbiology, Medical Faculty, Hacettepe University, Ankara, Turkey; ^16^Mt Sinai Hospital & Public Health Laboratories, Toronto, Ontario, Canada; ^17^Menzies School of Health Research, Charles Darwin University, Australia; ^18^Centre for Integrative Genomics, University of Lausanne, Switzerland; ^19^Department of Epidemiology, Center for Communicable Disease Dynamics, Harvard School of Public Health; ^20^Department of Medicine, Addenbrookes Hospital, University of Cambridge, United Kingdom

**Keywords:** pneumococcal isolates, whole-genome sequencing, comparative genomics, integrative conjugative elements, antibiotic nonsusceptibility

## Abstract

The surrounding capsule of *Streptococcus pneumoniae* has been identified as a major virulence factor and is targeted by pneumococcal conjugate vaccines (PCV). However, nonencapsulated *S. pneumoniae* (non-Ec-*Sp*) have also been isolated globally, mainly in carriage studies. It is unknown if non-Ec-*Sp* evolve sporadically, if they have high antibiotic nonsusceptiblity rates and a unique, specific gene content. Here, whole-genome sequencing of 131 non-Ec-*Sp* isolates sourced from 17 different locations around the world was performed. Results revealed a deep-branching classic lineage that is distinct from multiple sporadic lineages. The sporadic lineages clustered with a previously sequenced, global collection of encapsulated *S. pneumoniae* (Ec-*Sp*) isolates while the classic lineage is comprised mainly of the frequently identified multilocus sequences types (STs) ST344 (*n* = 39) and ST448 (*n* = 40). All ST344 and nine ST448 isolates had high nonsusceptiblity rates to β-lactams and other antimicrobials. Analysis of the accessory genome reveals that the classic non-Ec-*Sp* contained an increased number of mobile elements, than Ec-*Sp* and sporadic non-Ec-*Sp*. Performing adherence assays to human epithelial cells for selected classic and sporadic non-Ec-*Sp* revealed that the presence of a integrative conjugative element (ICE) results in increased adherence to human epithelial cells (*P* = 0.005). In contrast, sporadic non-Ec-*Sp* lacking the ICE had greater growth *in vitro* possibly resulting in improved fitness. In conclusion, non-Ec-*Sp* isolates from the classic lineage have evolved separately. They have spread globally, are well adapted to nasopharyngeal carriage and are able to coexist with Ec-*Sp.* Due to continued use of PCV, non-Ec-*Sp* may become more prevalent.

## Introduction

*Streptococcus pneumoniae* is an important human pathogen usually surrounded by a polysaccharide capsule which is considered to be a major virulence factor. Some isolates do not possess a capsule and are referred to as nonencapsulated *S. pneumoniae* (non-Ec-*Sp*). Although generally considered less virulent than encapsulated strains, non-Ec-*Sp* are also isolated from sterile sites and make up 10% of those isolated from the nasopharynx (Finland and Barnes 1977; Carvalho et al. 2003). This may be an underestimate as non-Ec-*Sp* form small rough colonies on agar plates which might be overlooked. Furthermore, the proportion of pneumococci colonizing the nasopharynx of young children that are non-Ec-*Sp* may be increasing in the current era of vaccines directed against polysaccharide capsules (Sá-Leão et al. 2009).

Some non-Ec-*Sp* clones have been repeatedly isolated worldwide, particularly sequence types (STs) ST344 and ST448 indicating intercontinental spread that may reflect an adaptive advantage in transmission. These types are associated with outbreaks of conjunctivitis (Martin et al. 2003; Porat et al. 2006). non-Ec-*Sp* are also particularly associated with cocolonization with other pneumococci in the nasopharynx, offering frequent opportunities for recombination (Brugger et al. 2009). Finally, non-Ec-*Sp* may be repositories of antibiotic resistance genes which could be transferred to encapsulated pneumococci (Hauser et al. 2004; Chewapreecha et al. 2014).

Previous studies of encapsulated *S. pneumoniae* (Ec-*Sp*) have attempted to define the pan genome of the species, but the gene content of non-Ec-*Sp* has not been systematically studied (Obert et al. 2006). Genes exclusively and consistently present in non-Ec-*Sp* (i.e., part of the core genome in this group, but not in other pneumococci) may be an adaptation for colonization in the absence of capsule. Sequencing of the capsule region of non-Ec-*Sp* has revealed the presence of coding sequences (CDS) named *aliB*-like open reading frame (ORF)1 and ORF2 due to their homology to *aliB* which encodes the substrate-binding protein of an ABC transporter for branched-chain amino acids (Hathaway et al. 2004). *AliB-like* ORF1 and ORF2 have recently been shown to play a role in sensing and responding to peptides in the environment (Claverys et al. 2000; Hathaway et al. 2014). Both *AliB* and *AliB-like* ORF2 have been reported to play a role in colonization (Kerr et al. 2004; Hathaway et al. 2014). *AliB-like* ORFs have also been found in the capsule region of the closely related species *Streptococcus mitis*, *Streptococcus oralis*, and *Streptococcus pseudopneumoniae* as well as preceding the capsule genes in pneumococci of serotypes 25F and 38 (Bentley et al. 2006). Salter et al. (2012) found another novel gene, a putative surface-anchored protein in place of capsule genes which was designated *nspA*. The same gene was called *PspK* and was revealed to increase adherence to epithelial cells within other studies (Park et al. 2012; Keller et al. 2013).

In this study, we sought to employ whole-genome sequence (WGS) analysis to define the phylogenetic population structure of non-Ec-*Sp* and their relationship to Ec-*Sp.* We aimed to reveal whether non-Ec-*Sp* are members of a separate lineage from Ec-*Sp* and whether there are non-Ec-*Sp*-specific genes which might shed light on the mechanism of colonization and which we investigated by *in vitro* experiments. We were also interested in the possible role of non-Ec-*Sp* as reservoirs of antibiotic resistance and so determined the resistance of the strains to a panel of antibiotics and investigated the alleles of relevant genes.

## Materials and Methods

### Sequencing of a Reference Isolate

We sequenced the non-Ec-*Sp* isolate 110.58 (ST344) on the Pacific Biosciences (PacBio) RS II platform to obtain a fully assembled reference genome. Sequence assembly yielded one major contig of 2.29 Mb and three small contigs at low coverage. The major contig was subsequently closed by running a second assembly. This was used as the reference sequence for subsequent analysis and has the accession number CP007593. The reference genome 110.58 consisted of 2,287,774 bp (39.7% GC content). Gene prediction and annotation was performed using Prodigal (version 2.60) and RAST server (http://rast.nmpdr.org/, last accessed December 1, 2014), respectively. There were 2,246 predicted CDS. Further details are given in the supplementary methods, Supplementary Material online.

### Strain Selection and Identification of Global non-Ec-Sp Isolates and Antimicrobial Susceptibility Tests

Further non-Ec-*Sp* were sourced from 17 different geographical locations including Europe (Portugal, Poland, Scotland, the Netherlands, and Switzerland), Africa (Kenya and Ghana), Asia (Turkey and Thailand), Australia, and the Americas (Peru, Massachusetts, Alaska and Canada). The isolates were checked for the presence of contamination by growing on blood agar plates and colony purified. Subsequently, optochin susceptibility was determined for all isolates followed in case of optochin resistance, by testing of bile solubility. In addition, the absence of *cpsA* was verified by real time polymerase chain reaction using the primers as described (Hathaway et al. 2007). The optochin susceptibility and bile solubility tests are consistent with the species assignment of *S. pneumoniae*, whereas the absence of *cpsA* is consistent with the presence of a true, nonencapsulated isolate, that is, an isolate without the first gene of the capsule operon. Antimicrobial susceptibility tests were performed for all isolates except the nine from Australia for which only DNA was available. Disc diffusion and minimal inhibitory concentrations (MIC) for penicillin testing was performed using standardized methods (Clinical and Laboratory Standards Institute 2012). The double-disc diffusion test (“*D*-test”) for erythromycin and clindamycin was performed as described previously (Asmah et al. 2009). In total, 216 non-Ec-*Sp* isolates were confirmed of which 131 were sequenced. The criteria for selection for sequencing were: 1) all ST344 and 448 isolates; 2) all isolates representing a unique geographical location, and 3) a variety of antibiotic profiles (table 1 and supplementary table S1, Supplementary Material online).

**Table 1:** Characteristics of Global non-Ec-*Sp* Isolates

	Classic Nonencapsulated			Sporadic Nonencapsulated (%)
	ST344 (%)	ST448 (%)	Other ‘classic’ (%)	
Total	38 (100)	40 (100)	11 (100)	42 (100)
“Capsule” (cps) genes				
*aliB*-like ORF1	38 (100)	40 (100)	11 (100)	17 (40)
*aliB*-like ORF2	38 (100)	40 (100)	11 (100)	18 (43)
*nspA*	0	0	0	11 (26)
“Real” *cps* genes	0	0	0	7 (17)
No genes	0	0	0	6 (14)
Antibiogram^a^				
Tetracycline, *r*	36 (95)	3 (8)	2 (18)	15 (36)
Levofloxacin, *r*	0	0	0	0
Vancomycin, *r*	0	0	0	0
Chloramphenicol, *r*	0	0	0	1 (2)
Trimethoprim–sulfamethoxazole, *r*	37 (97)	11 (28)	7 (39)	29 (69)
Erythromycin, *r*	36 (95)	0	0	7 (17)
Erythromycin, *i*	1 (3)	3 (8)	0	1 (2)
Clindamycin, cMLS_B,_	32 (84)	0	0	7 (17)
Clindamycin, iMLS_B_	4 (11)	0	0	0
Penicillin (MIC)				
<0.06 µg/ml	0	31 (78)	9 (82)	6 (14)
0.06–0.25 µg/ml	32 (84)	0	2 (18)	9 (21)
0.38–2 µg/ml	6 (16)	9 (23)	0	12 (28)
>2 µg/ml	0	0	0	2 (5)
Presence of resistance genes				
Presence of *tetM*	38 (100)	3 (8)	2 (18)	16 (38)
Presence of *ermB*	36 (95)	0	0	7 (17)
Presence of *mefE*	1 (3)	3 (8)	0	1 (2)
Geographical origin				
Portugal	20 (53)	2 (5)	3 (27)	5 (12)
Thailand		9 (23)		17 (40)
Switzerland	11 (29)	2 (5)		
Canada		9 (23)		2 (5)
Australia		1 (3)		8 (19)
Massachusetts	1 (3)	6 (15)	1 (9)	
Poland	3 (8)			3 (7)
Scotland	1 (3)	4 (10)		
Alaska	1 (3)	1 (3)	1 (9)	1 (2)
Netherlands		3 (8)	1 (9)	
Peru		1 (3)		2 (5)
Kenya		2 (5)	1 (9)	
India				3 (7)
Turkey	1 (3)		1 (9)	
Ghana			2 (18)	
Nepal			1 (9)	
Mongolia				1 (2)

^a^One ST448 and 8 “sporadic” isolates from Australia had no antibiogram; *r* (resistant) and *i* (intermediate) according to disc diffusion test and CLSI guidelines (Clinical and Laboratory Standards Institute 2010). Constitutively (cMLS_B_) macrolide–lincosamide–streptogramin B or inducible (iMLS_B_) phenotype. MIC, Minimal Inhibitory Concentration in μg/ml.

### WGS and Assembly for non-Ec-*Sp*

For WGS, multiplexed libraries were created and subsequent sequencing was performed on the Illumina HiSeq platform producing paired-end reads as described (Croucher et al. 2011). Eleven isolates did first not pass the quality control and were reisolated and resequenced (supplementary table S2, Supplementary Material online). Finally, all the Illumina reads of the 131 samples were then mapped to the reference genome 110.58. In total, a mean of 91.7% (range: 77.7–99.8%) reads were mapped to the reference genome. This resulted in a 10× genome coverage of 91.8% (range: 79.5–100%) (supplementary table S2, Supplementary Material online). De novo assembly was performed for all isolates using Velvet (Zerbino and Birney 2008).

### Calculation of the Core Clusters of Orthologous Groups and Diversity

For calculation of the core clusters of orthologous groups (COGs) of genes we additionally downloaded the assemblies from 44 Ec-*Sp* isolates previously published (Donati et al. 2010). This included the high quality assembled reference genome *S. pneumoniae* ATCC 700669 (Croucher et al. 2009). Subsequently, the contigs of the non-Ec-*Sp* and the Ec-*Sp* isolates were ordered using Mauve (Darling et al. 2010). For the Ec-*Sp* isolates, ATCC 700669 was used as a reference and for the non-Ec-*Sp* isolates the PacBioBiosciences assembly of 110.58 was used. Contigs that were shorter than 500 bp were excluded from the assemblies. The ordered contigs were concatenated. Gene prediction was again performed using Prodigal (version 2.60) included in the genome annotation pipeline Prokka (version 1.8; Hyatt et al. 2010). All protein sequences identified were compared with each other using BLASTP (version 2.2.27+) (Altschul et al. 1990). Only protein alignments with a minimal alignment length of ≥70%—with regard to the longer protein—and an alignment similarity of ≥70% were used to construct edges for the graph analysis using NetworkX (http://networkx.github.io, v1.7). Each protein of every isolate was used as node in the graph. Clusters that contain proteins found in all isolates represent the core genome. Presence and absence of genes compared with the reference genome 110.58 were plotted. integrative conjugative element (ICE) proteins were identified using the ICEberg database (Bi et al. 2012) and complete ICEs assembled using the reference genome 110.58.

As assembly errors may contribute to the variation in gene content, we compared the Illumina with the PacBio assembly of 110.58 to estimate the possible divergence. Within Illumina assemblies, we identified 15 genes in 14 COGs not present in the assembly received by PacBiosequencing (i.e., 0.72%). We therefore conclude that there was some divergence due to assembly errors but this would not affect the results significantly.

### Calculation of the Unique COGs within non-Ec-*Sp*

To define genes that uniquely appear in non-Ec-*Sp*, all the identified COGs were used. Each COGs was then marked as unique if it was present in ≥80 non-Ec-*Sp* but absent in all 44 Ec-*Sp*. For the newly defined region of diversity 2 (RD_2_*Sp*ST344), we additionally calculated the COGs which were present in ≥40 Ec-*Sp* but absent in all non-Ec-*Sp* (see table 2 for details). To enable annotation of the COGs, the sequence of the protein with the most edges to other genes in the clusters was extracted.

**Table 2:** Characteristics of COGs of the Region of Diversity (RD_2_*Sp*ST344)

SPN23F ID^a^	110.58 ID	No. of non-Ec-*Sp* with the gene (%)^b^	Number of Ec-*Sp* with the gene (%)^c^	Annotation COGs
NA	02261	95 (72)	0	hypothetical protein
NA	02262	93 (70)	0	putative protein encoded in hyper variable junctions of pilus gene clusters
NA	02263	95 (72)	0	hypothetical protein
NA	02264	105 (80)	0	site-specific tyrosine recombinase *xerd*
NA	02274	93 (70)	0	hypothetical protein
NA	02277	92 (70)	0	hypothetical protein
NA	02279	93 (70)	0	Beta-galactosidase
NA	02280	90 (68)	0	Immunoglobulin A1 protease precursor^d^
NA	02283	94 (71)	0	Outer membrane lipoprotein P4
21800	NA	0	42 (95)	arginine deiminase *arcA*
21890	NA	0	44 (100)	alcohol dehydrogenase, iron-containing; *medH*
21900	NA	0	44 (100)	L-fucose isomerase *fucI*
21990	NA	0	40 (91)	fuculokinase *fucK*
22000	NA	0	41 (93)	putative fucose phosphotransferase system

^a^Numbering according to (Croucher et al. 2009).

^b^COGs present ≥80 nonencapusulated *Streptococcus pneumoniae* (non-Ec-*Sp*) but completely absent in 44 published Ec-*Sp* (Donati et al. 2010).

^c^According to the collection of 44 published *Ec-Sp* (Donati et al. 2010). COGs present ≥40 Ec-*Sp* but absent in all non-Ec-*Sp* are included.

^d^Gene is annotated as a putative zinc metalloprotease (*zmpC*) by a recent study (Keller et al. 2013).

### Phylogenetic Tree Construction

Genes present as a single copy in every genome (132 non-Ec-*Sp* (including 110.58 PacBio), 44 Ec-*Sp*), with the identical nucleotide length were designated as “core.” The ORF of the 363 COGs were aligned separately using Clustal Omega (version 1.1.0; Sievers et al. 2011). The resulting alignment files were fused using a Python script. Out of the fused alignment file the phylogenetic tree was constructed using RaxML (version 7.2.8a, raxmlHPC-PTHREADS-SSE3 -m GTRGAMMA-# 50) (Stamatakis 2006). The resulting phylogeny file was visualized using Figtree.

### Analysis of ST344 and ST448 within Classical non-Ec-*Sp*

To analyze recent evolution of ST344 and ST448 isolates within the classical non-Ec-*Sp*, rates of single nucleotide polymorphism (SNP) and recombination were determined. To do this, whole-genome alignments were generated for 38 isolates of ST344 isolates and 40 of ST448. non-Ec-*Sp* 110.58 was used as a reference, and Illumina reads were mapped using SMALT resulting in two alignment files. Rates of recombination in ST448 and ST344 were calculated as the ratio of the number of homologous recombination events to the number of mutations (*r*/*m*) (Croucher et al. 2011; Chewapreecha et al. 2014). In this study, we used the arithmetic mean of *r*/*m* for a cluster, averaged from the *r*/*m* of each branch within a cluster as previously described (Croucher et al. 2011). The mean of the distribution of *r*/*m* for the cluster was then obtained. To produce phylogenetic trees from the whole-genome alignments for the isolates of the ST344 and ST448 clones, SNPs due to recombination were excluded.

### Adherence to Human Epithelial Cell Line Detroit 562 and Analysis of Planktonic Growth

As for measuring planktonic growth, 96-well microtiter polystyrene plates (Thermo Fisher Scientific, Denmark) were used for the isolates Switzerland 4, 11, Thailand 1 and 2, respectively. Wells were filled with a total of 192 μl of liquid chemically defined medium (CDM) supplemented with 5.5 mM glucose. Subsequently 8 μl (dilution = 1:25) of fresh bacterial suspension was added. Plates were sealed with parafilm, and OD_450nm_ was measured on an Enzyme Linked Immunosorbent Assay (ELISA) plate reader (THERMOmax Microplate Reader, Molecular Devices Corporation, California) every 30 min for 22 h using SOFTmax Pro 3.1.2. This also involved an essential 5 s of automatic shaking in the plate reader immediately before every measurement. Each experiment included three technical replicates and each experiment was performed three times.

For adherence assays, Detroit nasopharyngeal epithelial cells (ATCC-CCL-138) were cultured as described (Luer et al. 2011). Adherence experiments for media including 5.5 mM glucose were subsequently performed nine times (three times at three different time points) as recently described (Schaffner et al. 2014). Ordinary one-way Analysis of variance (ANOVA) was performed to reveal statistical significance among the four isolates chosen for the experiments (Switzerland 4, 11, Thailand 1 and 2).

## Results and Discussion

### Identification of “Classic” and “Sporadic” non-Ec-*Sp* by WGS

Whole genomic sequences were determined for a global collection of 131 non-Ec-*Sp* from 17 different geographical sites (table 1 and supplementary table S1, Supplementary Material online). Assembled genomes were annotated revealing a total of 1,148 COGs which were present in all 131 non-Ec-*Sp* genomes and therefore represent the non-Ec-*Sp* core genome. Genomes of recently published, global collection of 44 Ec-*Sp* were added to the data set reducing the number of core COGs to 858 (Donati et al. 2010).

**Fig. 1.—:**
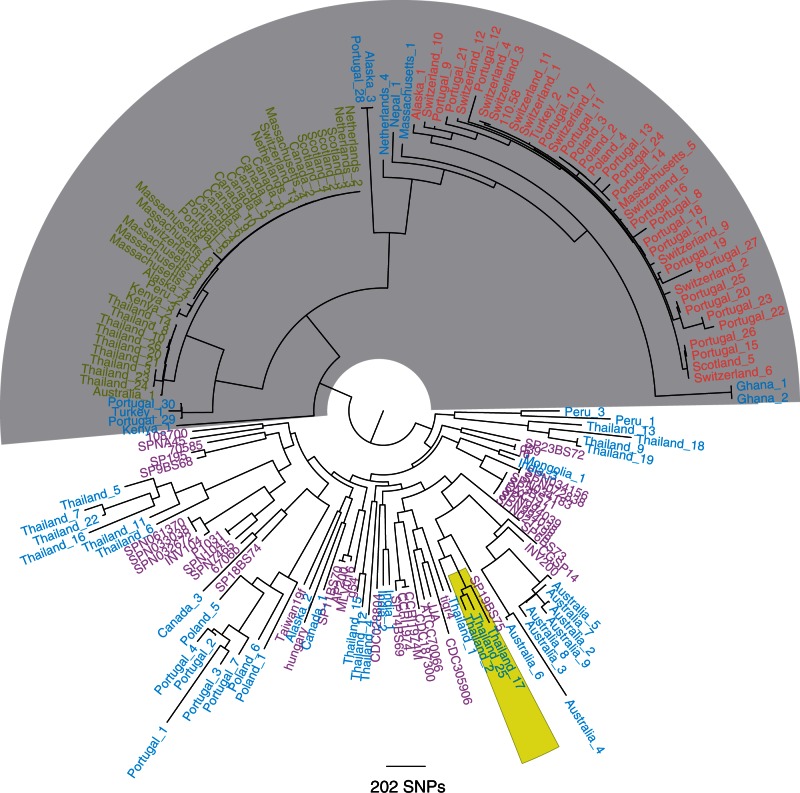
Core genome tree of 131 nonencapsulated and 44 encapsulated *S. pneumoniae*. One hundred thirty-one sequences of non-Ec-*Sp* isolates from 17 different countries or states of America were included (shown in red, green, and blue). The classic lineage of exclusively non-Ec-*Sp* (light gray) contains the isolates of ST448 (green) and ST344 (red). Additionally published encapsulated pneumococci (indicated in pink) (Donati et al. 2010) clustered within other non-Ec-*Sp* (blue). COGs that are present in every isolate as a single copy and, in addition, having the same sequence length were selected for building the tree (363 COGs). The maximum-likelihood phylogeny was generated using 24,342 polymorphic sites within a 278,778 bp codon alignment. Three isolates of the recently published BC3-NT clade are also indicated (yellow) (Chewapreecha et al. 2014).

Subsequently, only genes present in every isolate as a single copy were selected (764 COGs). In addition, all the genes which did not have the same length in all the isolates were discarded resulting in 363 COGs (fig. 1). By comparing SNPs in these 363 COGs, we identified a lineage of exclusively non-Ec-*Sp* which we have designated as the classic lineage (light gray background; fig. 1). Within this single lineage the isolates of ST344 and ST448 form distinct clades.

The remaining non-Ec-*Sp* were distinct from the classical lineage, clustered with Ec-*Sp*, and are part of multiple sporadic lineages. Therefore, the non-Ec-*Sp* of these lineages may have been encapsulated strains which lost the capsule “sporadically” by acquiring the *aliB*-like ORFs, *nspA* (Salter et al. 2012) or no additional genes (table 1). Isolates from the recently published BC3-NT lineage, highly prevalent among the isolates from a Thai refugee camp, clustered with one of the sporadic lineages (Chewapreecha et al. 2014; fig. 1).

The existence of a distinct lineage of non-Ec-*Sp* has been previously proposed on the basis of multilocus sequence typing (MLST) data (Hanage et al. 2006; Croucher et al. 2013) and is consistent with genome sequencing of a local sample collected in Massachusetts (Croucher et al. 2013). Here, we show that WGS of a global collection of isolates confirms the existence of a divergent non-Ec-*Sp* group containing ST344 and ST448 but also other STs (supplementary table S1, Supplementary Material online).

ST344 and ST448 may be particularly well adapted to colonization of the nasopharynx and conjunctiva. All the Canadian and Scottish isolates of ST448 were isolated from the eye, in concordance with the work of other groups who associated ST448 with outbreaks of conjunctivitis (Porat et al. 2006). All other ST448 isolates and all ST344 isolates were isolated from the nasopharynx.

### Classic and Sporadic non-Ec-*Sp* Lineages by Accessory Genome Diversity Comparison

**Fig. 2.—:**
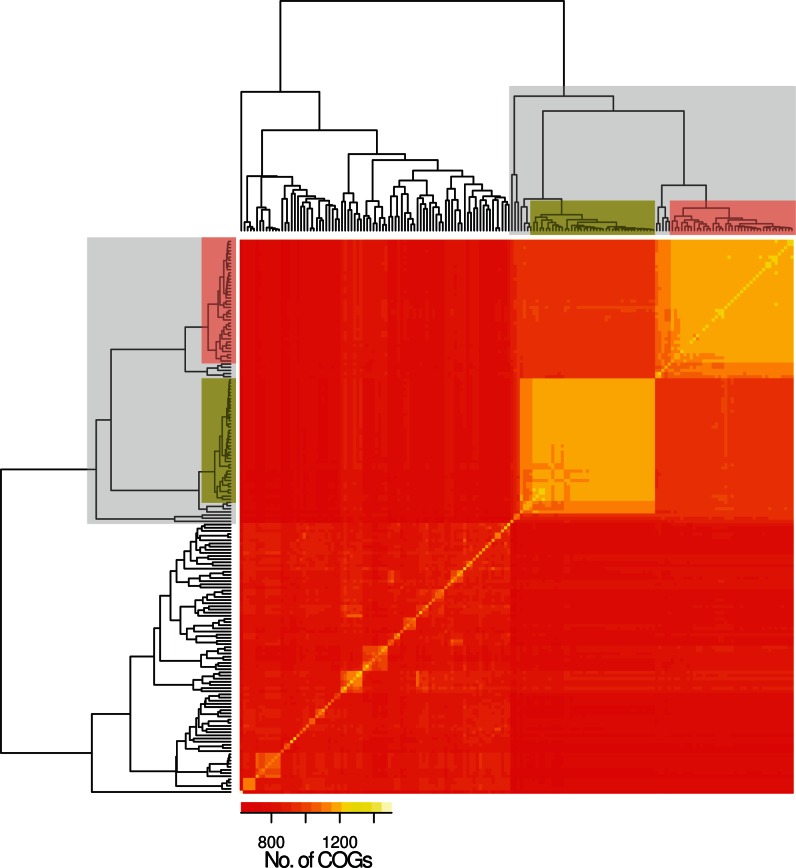
Accessory genome diversity of 131 nonencapsulated and 44 encapsulated *S. pneumoniae*. The classic lineage of exclusively non-Ec-*Sp* is indicated (light gray) and contains the isolates of ST448 (green) and ST344 (red). The remaining isolates are sporadic non-Ec-*Sp* and Ec-*Sp*. To construct the input array data all isolates were compared pairwise. Of each pair the COGs (70% coverage, 70% similarity) which are not part of the core-genome and are present in both isolates were counted. The isolates in the x and y axes are ordered according to the Euclidean distance constructed dendrogram.

In addition to SNPs of core COGs, we investigated whether analysis of distinct gene content in the accessory genome supported the finding of a separate classic non-Ec-*Sp* lineage. For this, we determined the accessory genome for each non-Ec-*Sp* and Ec-*Sp* individually and performed pairwise comparisons which confirmed the separation of classic versus sporadic non-Ec-*Sp* revealed by analysis of the SNPs of the core COGs (fig. 2). Again, the clones ST344 and ST448 were found in the classic lineage (fig. 2).

**Fig. 3.—:**
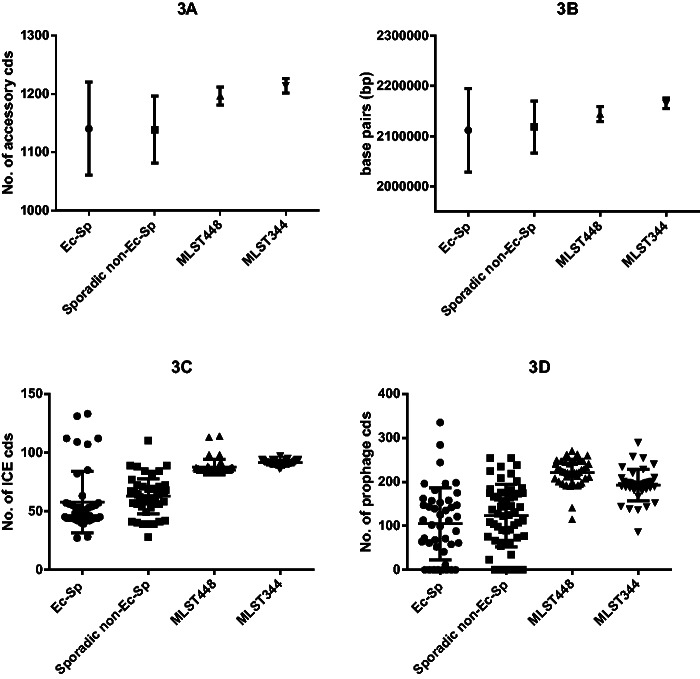
Size and composition of accessory genome. The total number of accessory genes (*A*) of each isolate was determined by number of homologous groups (70% coverage, 70% similarity) which are present in the specific strain and not part of the core genome. The total genome sizes of all isolates are indicated (*B*). To detect genes of possible ICEs (*C*) in the different isolates, all CDS of each strain were aligned to the ICEberg database [1] (March 2014) using BLASTP (version 2.2.28+) [2]. Every CDS that showed homology (70% coverage, 70% similarity) to at least one ICE-protein was assumed to be ICE related. To determine the number of proteins that originate by phages (*D*), the genomes were scanned for possible prophage region using PhiSpy (version 2.2) [3]. All the CDS that lie in the region of a predicted prophage region were counted for each isolate separately. Means and SD (standard deviation) are indicated (A-D).

We also found that the accessory genomes and the genome sizes of classic non-Ec-*Sp* are at the upper end of the spectrum as compared with the sporadic non-Ec-*Sp* and the Ec-*Sp* (fig. 3*A* and *B*). This observation also holds true if only ICE and prophage proteins within the accessory genomes are analyzed (fig. 3*C* and *D*). Therefore, classic non-Ec-*Sp* may act more likely as a gene reservoir as compared with either sporadic non-Ec-*Sp* or Ec-*Sp* strains.

### Characteristics of COGs within Classic non-Ec-*Sp* Which Are Absent in Ec-*Sp*

**Fig. 4.—:**
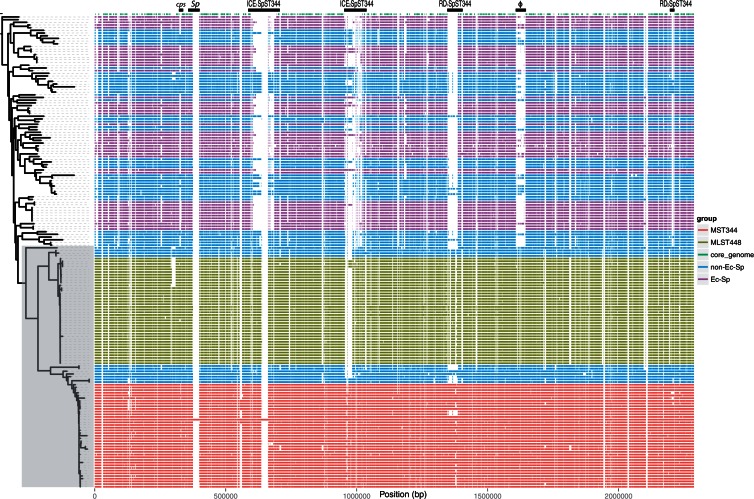
Presence and absence of CDS of nonencapsulated and encapsulated *S. pneumoniae* compared with an ST344 reference genome. Each row illustrates a specific isolate genome with the presence or absence of CDS as defined in the reference genome (110.58). The rows are ordered by the phylogeny of the isolates (indicated on the left). The *x* axis represents the genomic position of the CDS in the genome of 110.58. The figure was constructed using R (version 3.1.0 alpha) with the “ggplot2” package. Two large surface proteins (*Sp*) were not correctly assembled in the illumina sequenced isolates and are therefore lacking. The reference genome (110.58) contains the genes *aliB*-like ORF1 and ORF2 at the cps (capsule) operon (Hathaway et al. 2004) which were absent and replaced by cps genes in Ec-*Sp*. RD, region of diversity; cps, capsule operon; ϕ, prophage.

In order to investigate whether non-Ec-*Sp* have their own distinctive gene content, we examined COGs unique to non-Ec-*Sp* but absent in the published Ec-*Sp* (Donati et al. 2010; supplementary table S3, Supplementary Material online). We found a total of 116 COGs which were present in ≥80% non-Ec-*Sp* but absent in all 44 Ec-*Sp* (supplementary table S4, Supplementary Material online). The location of these and the core COGs as compared with the reference genome 110.58 were plotted in figure 4.

As expected, 6/116 COGs were identified as unique for ≥80 non-Ec-*Sp* within the capsule region. non-Ec-*Sp* have no capsule but other genes have been described as being characteristic of this region in nonencapsulated isolates. Furthermore, 13/116 COGs were identified within prophage regions. Although three COGs belonged to a prophage remnant, the remaining COGs belonged to a complete phage which was inserted within exactly the same location of 110.58 as is the φMM1 phage for *S. pneumoniae* ATCC 700669. Production and release of this new prophage in isolate 110.58 when the culture was allowed to progress to lysis was confirmed by electron microscopy and is characteristic for non-Ec-*Sp* isolates as we found this prophage neither in the sporadic non-Ec-*Sp* nor in the Ec-*Sp* of this study (supplementary fig. S1, Supplementary Material online). Seventeen COGs were dispersed within the genome, while the majority of COGs (*n* = 80) were found within two major regions of differences (RDs) and ICEs (fig. 4 and supplementary table S4, Supplementary Material online).

### Two Complete ICEs Are Present within Classic non-Ec-*Sp*

**Fig. 5.—:**
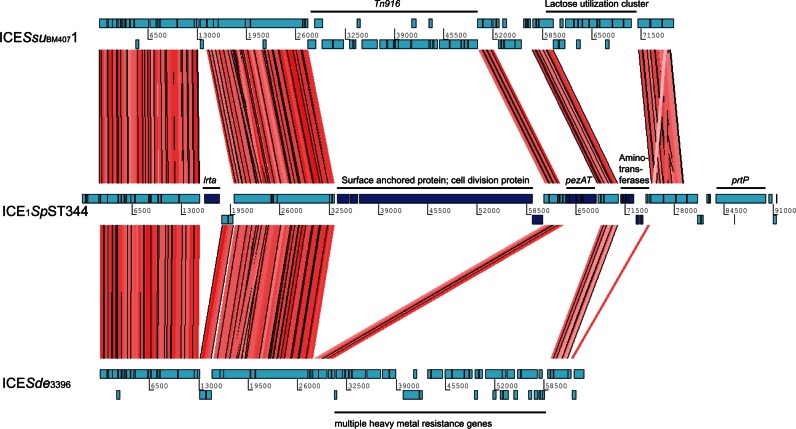
Genetic organization and nucleic acid alignment of ICEs. Artemis comparison tool (ACT) was used for the nucleic acid alignment of the ICE*Ssu*_BM4407_1 (*S. suis)*, ICE_1_*Sp*ST344 and ICE*Sde*_3396_ (*Streptococcus dysagalagticae*). Individual CDS are indicated in light blue. Conserved CDS between the three ICEs are indicated by red shading. CDS which were unique for ICE_1_*Sp*ST344 are indicated in dark blue. Tn, transposon; *pezAT*, toxin–antitoxin genes: *lrtA*, reverse transcriptase A; *prtP*, PII-type proteinase precursor.

**Fig. 6.—:**
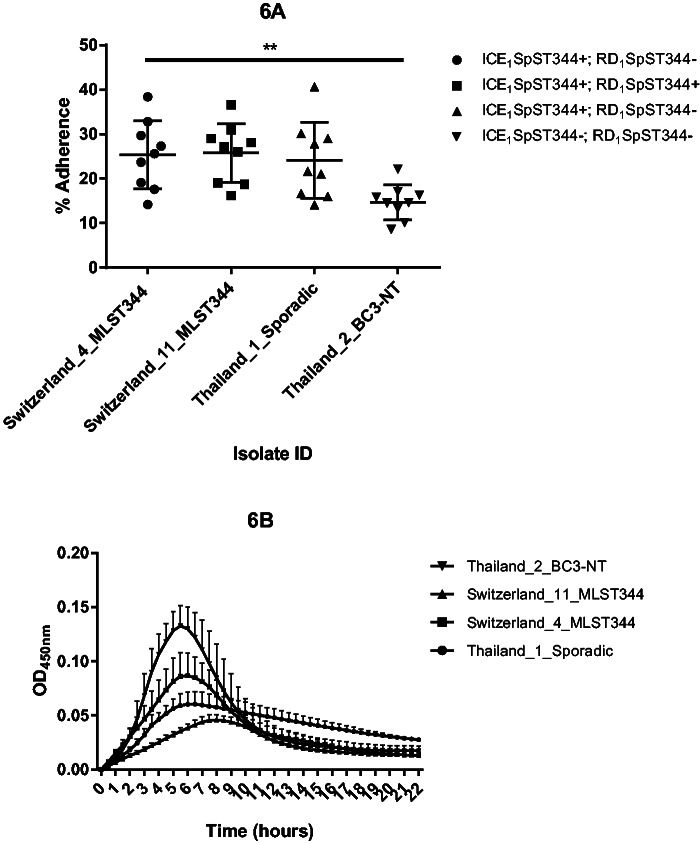
Adherence of *S. pneumoniae* to human epithelial cells (*A*) and in vitro growth (*B*). Detroit nasopharyngeal epithelial cell lines were exposed to non-Ec-*Sp* containing or lacking ICE_1_*Sp*ST344 and RD_1_*Sp*ST344, respectively (*A*). Adherence was determined at 30 min and calculated as the proportion of recovered bacteria to the inoculum. Experiments were repeated three times (three times on three different days: Indicated are mean and SD (standard deviation)). Swiss and Thai strains were classic and sporadic non-Ec-*Sp*, respectively. For the nine adherence experiments, ordinary one-way ANOVA resulted in *P* = 0.0052. See text for details. MLST, multilocus sequence type. Isolates of the BC3-NT lineage have been recently defined (Chewapreecha et al. 2014). Measurement of planktonic growth was done in 96-well microtiter polystyrene plates and OD450nm was measured on an ELISA plate reader every 30 min for 22 h (*B*). Each experiment included three technical replicates and each experiment was performed three times.

Among the unique COGs within ICEs, there were 23 within ICE_1_*Sp*ST344. Having the reference genome 110.58 allowed us to assemble these ICE proteins to a complete ICE (fig. 5). Though not yet described in *S. pneumoniae*, similar ICEs (∼80% nt identity to ICE_1_*Sp*ST344) were described in *Streptococcus suis* (ICE*Ssu*) and *S. dysgalactiae* (ICE*Sde*3396) (Davies et al. 2009; Holden et al. 2009) (fig. 5). As for the cargo genes within ICE_1_*Sp*ST344of non-Ec-*Sp*, a large surface protein and an epsilon toxin-zeta antitoxin system (*pezAT*) was identified. *PezAT* has been characterized in terms of structure and function and its presence may result in outcompeting other bacteria during colonization (Chan et al. 2012). However, COGs of ICE_1_*Sp*ST344 were also found within sporadic non-Ec-*Sp* (fig. 4). As the two lineages are phylogenetically different, the ICE_1_*Sp*ST344 may have been acquired independently through conjugation or possibly transformation. We therefore hypothesized that COGs of these ICEs could be important for the life cycle of non-Ec-*Sp.* Indeed, isolates containing ICE_1_*Sp*ST344 revealed an approximate 2-fold greater adherence to Detroit nasopharyngeal epithelial cells (*P* = 0.005; fig. 6*A*). However, isolates of the BT3-NT lineage were isolated frequently in the refugee camp on the Thailand–Myanmar border despite lacking ICE_1_*Sp*ST344 (fig. 6*A*), but showed improved growth (fig. 6*B*). In contrast, the isolate lacking both, ICE_1_*Sp*ST344 and RD_1_ showed poor growth in CDM supplemented with 5.5 M of glucose (fig. 6*B*). This may mean that having many mobile elements may result in a fitness cost and that there is a careful balance between fitness, improved adherence and antibiotic resistance.

There were a further ten unique COGs identified in an additional ICE, called ICE_2_*Sp*ST344 (fig. 4). In contrast to ICE_1_*Sp*ST344, ICE_2_*Sp*ST344 is not unique for non-Ec-*Sp* as it has been indentified, for example, in *S. pneumoniae* Spain23F ST81 (Croucher et al. 2009). However, although ICE_2_*Sp*ST344 is somewhat similar to the ICE*Sp*23FST81, an additional putative lantibiotic transporter protein has been found within non-Ec-*Sp* as previously described for an “ancestral” *S. pneumoniae* (ICE*Sp*PN1) strain (Wyres et al. 2013).

Finally, two additional, unique COGs with unknown function were also identified within the Pneumococcal Pathogenicity Island 1 (PPI-1) which has been described as ICE-derived genomic island (Croucher et al. 2009). As characteristic for ICE_1_*Sp*ST344 and ICE_2_*Sp*ST344, the PPI-1 also possesses an epsilon toxin-zeta antitoxin system.

### Two Major Regions of Differences (RD_1_*Sp*ST344 and RD_2_*Sp*ST344) Are Uniquely Present within non-Ec-*Sp*

The remaining COGs unique for non-Ec-*Sp*, were identified within two major regions of differences (RD_1_*Sp*ST344 and RD_2_*Sp*ST344). RD_1_*Sp*ST344 contains 35 COGs for the majority of non-Ec-*Sp* upstream of SPN23F08840 (translation initiation factor IF-3) which are absent in all Ec-*Sp* isolates (fig. 4). More than half of the COGs (20; 56%) are annotated as hypothetical protein with unknown function. Among those remaining are mobilization and conjugation proteins indicating that the genes of RD_1_*Sp*ST344 are part of a mobile element. The functional importance of RD_1_*Sp*ST344 within non-Ec-*Sp* has yet to be revealed. However, the presence of RD_1_*Sp*ST344 had no influence on the adherence potential of *S. pneumoniae* to Detroit nasopharyngeal epithelial cells (fig. 6*A*).

RD_2_*Sp*ST344 is situated between the ribosomal protein L33 (SPN23F21670; 110.58_2260) and a zinc ABC transporter (SPN23F2201; 110.58_2284). In contrast to RD_1_*Sp*ST344, RD_2_*Sp*ST344 is present in non-Ec-*Sp* and Ec-*Sp* but there is considerable diversity. Among the genes which are uniquely present within non-Ec-*Sp* there is a beta galactosidase, an outer membrane lipoprotein and a putative zinc metalloprotease (table 2). These genes replace an arginine deaminase system (ADS) which is found within the Ec-*Sp* (table 2). ADS is thought to influence the arginine metabolism (Kloosterman and Kuipers 2011) and genetic inactivation of the ADS affects colonization and dissemination in mice (Schulz et al. 2013).

### High Antibiotic Resistance within non-Ec-*Sp*

An alternative hypothesis for the global success of ST344 and ST448 is a high antibiotic resistance in these clones. Therefore, 123 of the 131 isolates were tested for susceptibility to the main antibiotic classes (table 1). The three major genes which determine β-lactam susceptibility or resistance are the penicillin-binding proteins found on either side of the “capsule” locus (*pbp2x* and *pbp1a*) and elsewhere in the genome (*pbp2b*). Therefore, we analyzed the sequences of all three *pbp* genes to determine allelic combinations associated with increased MIC toward penicillin (supplementary figs. S2–S4, Supplementary Material online).

Overall, the majority of ST448 and other classic non-Ec-*Sp* isolates were fully susceptible to penicillin (table 1). In contrast, the classic non-Ec-*Sp* isolates of ST344 had a MIC of at least 0.06 µg/ml but not more than 2.0 µg/ml for penicillin (table 1). This may indicate a well-adapted balance between a basic tolerance toward penicillin without having the cost for fitness loss as shown recently for isolates with very high penicillin resistance (Trzcinski et al. 2006).

With regard to non-β-lactam antibiotic resistance, 95% of ST344 isolates (*n* = 36) were resistant toward both tetracycline and erythromycin (table 1). The presence of *tet*(*M*) was confirmed in all the tetracycline nonsusceptible isolates. However, three additional isolates carrying the *tet*(*M*) gene were found to be susceptible due to disruption of the gene leading to a frameshift mutation.

As for macrolide resistance, 36 isolates of the classic (ST344) and 7 of the sporadic lineages were fully resistant due to the presence of the *ermB* rRNA methylase. Four isolates initially presented erythromycin resistance but clindamycin susceptibility despite *ermB* being present. Subsequent erythromycin–clindamycin *D*-test revealed inducible expression of the resistance phenotype for these isolates. Additionally, five isolates presented intermediate erythromycin resistance due to the presence of the *mel-mef* efflux pump. The presence of the *mel-mef* efflux pump is normally not as strongly associated with sequence clades, an observation consistent with our data (Croucher et al. 2013; table 1). The *ermB* gene has been shown to be carried by the *Tn*917 or Omega resistance cassettes (Croucher et al. 2013). The same was true for the non-Ec-*Sp* isolates for which the Tn917 and/or Omega resistance cassettes were identified within ICE_2_*Sp*ST344 (fig. 4).

As for sulfa drugs, ST448 isolates were generally susceptible to trimethoprim–sulfamethoxazole with the exception of those from Thailand (*n* = 9) and Kenya (*n* = 2). Within these, resistance-conferring mutations were detected in the genes *dyr* (encoding dihydrofolate reductase) and *folP* (dihydropteroate synthase). For ST344 all but the oldest isolate of the collection (Alaska 1 from 1998) were resistant to sulfa drugs (table 1) indicating a worrying evolution of ST344 toward high antibiotic resistance.

### Evolutionary Rates within ST344 and ST448

Previous studies revealed that *S. pneumoniae* recombines at different rates across different lineages. In particular, it has been found that a specific NT-lineage (BC3-NT) identified in a refugee camp on the Thailand–Myanmar border had very high frequencies of uptake and donation of DNA fragments compared with encapsulated lineages (Chewapreecha et al. 2014). However, as BC3-NT strains clustered with the sporadic non-Ec-*Sp* isolates within our global collection (fig. 1), we wondered whether the classic ST344 and 448 had similar recombination rates to BC3-NT. Furthermore, we were interested in analyzing whether the hot spot for recombination differs from those previously published within different lineages. In order to determine recombination rates, whole-genome alignments were generated for ST344 and ST448 isolates using the PacBio sequenced genome 110.58 as a reference. We then calculated the rates of recombination in ST344 and ST448 as the ratio of the number of homologous recombination events to the number of mutations (*r*/*m*) according to previously described methods (Croucher et al. 2011; Chewapreecha et al. 2014). The estimates for the *r*/*m* by recombination event per mutation were 0.18 (95% CI 0.12–0.26) and 0.15 (95% CI 0.02–0.28) for the ST344 and ST448 isolates, respectively. These values are lower than for the recently described, sporadic non-Ec-*Sp*, BC3-NT for which *r*/*m* was 0.3 (Chewapreecha et al. 2014) and approximately similar as for multidrug resistant Ec-*Sp* PMEN14 (0.17), PMEN1 (0.1), and PMEN2 (0.17) (Croucher et al. 2013, 2014). The low *r*/*m* values within ST344 and ST448 are surprising as it is generally assumed that the absence of the capsule as a physical barrier facilitates DNA exchange (Chewapreecha et al. 2014). However, the low values suggest that the classic non-Ec-*Sp* clones ST344 and ST448 are stable, fit and well adapted even though they lack the major virulence factor of the capsule.

### Recombination Hotspots within ST448 and ST344

Despite a general absence of recombination in ST448, frequent recombination sites were detected for the isolates from Australia (*n* = 1) and the refugee camp on the Thailand–Myanmar border (*n* = 8; supplementary fig. S5, Supplementary Material online). A driving force for the latter is probably the high antibiotic consumption within the camp as the recombination hotspots are *pbp2x*, *pbp2b*, and *pbp1a*, genes involved in penicillin resistance. Recombination within these genes was also detected within the worldwide ST344 collection, though the evolution went the “opposite” way, that is, toward gaining antibiotic susceptibility (supplementary fig. S6). The four outgroup isolates of this clone (Portugal 21, Switzerland 10, Portugal 9, Alaska 1) had a mean MIC for penicillin of 0.6 µg/ml (±0.2 µg/ml) (supplementary table S1, Supplementary Material online). Of the remaining ST344 isolates, all but two had lower penicillin resistance. This could be the result of opposing selection pressures: antibiotic-mediated selection versus the cost of acquiring resistance on strain fitness. However, as the number of sequenced isolates was limited, this inference may change with further sequencing. Penicillin-resistant strains have been shown previously to be less fit than susceptible strains in the absence of penicillin (Trzcinski et al. 2006). For the ST448 isolates, all but nine were fully susceptible to penicillin (eight isolates from Thailand and one from Australia; table 1). It is possible that there is a higher antibiotic pressure present in these two settings and therefore an expansion of a resistant subclone from within the susceptible lineage. Further hotspots of recombination affect choline-binding proteins like *lytB* (supplementary figs. S5 and S6, Supplementary Material online).

In conclusion, due to continued use of pneumococcal conjugate vaccines which target the pneumococcal capsule, non-Ec-*Sp* may become more prevalent in the future. This is why a thorough understanding of the types of non-Ec-*Sp* and their importance is urgently needed. Performing WGS of a global collection of non-Ec-*Sp* we clearly showed a distinct lineage of classical non-Ec-*Sp*. This lineage is dominated by, but not restricted to, ST344 and 448. The other non-Ec-*Sp* named here as “sporadic” cluster with encapsulated strains. Compared with Ec-*Sp*, classic non-Ec-*Sp* have a very high number of mobile elements (e.g., ICEs) resulting in an increased genome size and adherence to epithelial cells but a lowered potential of planktonic growth as shown in *in vitro* experiments.

## Supplementary Material

Supplementary methods, figures S1–S6, and tables S1-S4 are available at *Genome Biology and Evolution online* (http://www.gbe.oxfordjournals.org/).

Supplementary Data
