# Supplementary material for: Global Phylogenomic Analysis of Nonencapsulated Streptococcus pneumoniae Reveals a Deep-Branching Classic Lineage That Is Distinct from Multiple Sporadic Lineages
Source: Genome Biol Evol. 2014 Dec 24;6(12):3281–94. doi: 10.1093/gbe/evu263 (PMC4986459; doi:10.1093/gbe/evu263)
Supplement: Supplementary Data [file supp_6_12_3281__index.html]

Global phylogenomic analysis of nonencapsulated Streptococcus pneumoniae reveals a deep-branching classic lineage that is distinct from multiple sporadic lineages — Global Phylogenomic Analysis of Nonencapsulated Streptococcus pneumoniae Reveals a Deep-Branching Classic Lineage That Is Distinct from Multiple Sporadic Lineages — Supplementary Data 

# Global Phylogenomic Analysis of Nonencapsulated *Streptococcus pneumoniae* Reveals a Deep-Branching Classic Lineage That Is Distinct from Multiple Sporadic Lineages

## Supplementary Data

files

**Files in this Data Supplement:**

- Supplementary Data - xlsx file
- Supplementary Data - docx file
