# Supplementary material for: Global Phylogenomic Analysis of Nonencapsulated Streptococcus pneumoniae Reveals a Deep-Branching Classic Lineage That Is Distinct from Multiple Sporadic Lineages
Source: Genome Biol Evol. 2014 Dec 24;6(12):3281–94. doi: 10.1093/gbe/evu263 (PMC4986459; doi:10.1093/gbe/evu263)
Supplement: Supplementary Data [file supp_evu263_Supplementary_text_figures.docx]

**Supplemental Material**

**Supplementary methods**

**Whole genome assembly of a reference genome of ST 344**

High molecular weight DNA from *Streptococcus pneumoniae* was sheared in a Covaris g-TUBE (Covaris, Woburn, MA, USA) to obtain 20 kb fragments. After shearing the DNA size distribution was checked on a Fragment Analyzer (Advanced Analytical Technologies, Ames, IA, USA). 5 µg of the sheared DNA was used to prepare a SMRTbell library with the Pacific Biosciences DNA Template Prep Kit 2.0 (Pacific Biosciences, Menlo Park, CA, USA) according to the manufacturer's recommendations. 1 µg of the resulting library was size selected on a BluePippin system (Sage Science, Inc. Beverly, MA, USA) for molecules larger than 7 kb. The recovered library was sequenced on one SMRT cell with P4/C2 chemistry and MagBeads on a Pacific Biosciences RSII system (Pacific Biosciences, Menlo Park, CA, USA) at 180 min movie length. Sequencing yielded 70 348 post filter reads corresponding to 460 Mb with a mean read length of 6542 bases. 50% of bases were in reads longer than 7900 bp.

**Genome assembly, gene prediction and annotation of reference genome 110.58**

For the genome assembly, the Pacific Biosciences module "RS_HGAP_Assembly.2" in SMRTpipe version v2.1.1 was used. The seed read length cutoff (reads which after error correction and trimming, are used for Celera assembler) was set at 12 200 bp. The assembly yielded one major contig of 2291 Mb and 3 small contigs at low coverage. The major contig was closed by running a second assembly using Pacific Biosciences module "RS_HGAP_Assembly.1" in SMRTpipe version v2.0.1 was used. By this the origin of assembly was at another location, so the contigs could be closed by manually combining the two assemblies. Gene prediction was performed using Prodigal (version 2.60), which was used in the genome annotation pipeline Prokka ([Hyatt, et al. 2010](#_ENREF_4)). For the identification of ICE-regions within the Non-Ec-Sp 110.58 proteins of ICEberg were aligned with the complete sequence of 110.58 ([Bi, et al. 2012](#_ENREF_1)). As for the detection of phage regions, the genome was annotated using the RAST server (http://rast.nmpdr.org/) and followed by identifying pro-phages using the PHAST web-interface (http://phast.wishartlab.com/)

**References**

Bi D, et al. 2012. ICEberg: a web-based resource for integrative and conjugative elements found in Bacteria. Nucleic Acids Res. 40:D621-626.

Croucher NJ, et al. 2011. Rapid pneumococcal evolution in response to clinical interventions. Science. 331:430-434.

Denapaite D, et al. 2010. The genome of Streptococcus mitis B6--what is a commensal? PLoS One. 5:e9426.

Hyatt D, et al. 2010. Prodigal: prokaryotic gene recognition and translation initiation site identification. BMC Bioinformatics. 11:119.

**Legends for supplementary tables**

**Supplementary Table 1**: Accession numbers and antibiogramm for a global collection of 131 Non-Ec-*Sp* from 17 different geographical sites.

**Supplementary Table 2**: Overview of mapping of reads to the reference genome 110.58 (Sequence type 344).

**Supplementary Table 3:** Presence and absence of genes of the pan genome. Illustrated are genes present overall but also in Non-Ec-*Sp* (study_strains) and in Ec-*Sp* (reference_strains). The annotation, position and orientation according to reference genome 110.58 are illustrated, too.

**Supplementary Table 4**: Cluster of orthologous genes (COGs) which are present in ≥ 80% Non-Ec-*Sp* (study_strains) but absent in all 44 Ec-*Sp* (reference_strains).

**Legends for supplementary figures**

**Supplementary Figure 1**: Electron micrograph of the phage isolated from the nonencapsulated strain 110.58 (ST344). Phage was derived as described ([Denapaite, et al. 2010](#_ENREF_3)).

**Supplementary Figure 2: Phylogenetic tree of *pbp1a*.** The sequence clusters (ST344, ST448 and other), geographical location and minimal inhibitory concentrations (MICs) are indicated.

**Supplementary Figure 3: Phylogenetic tree of *pbp2b*.** The sequence clusters (ST344, ST448 and other), geographical location and minimal inhibitory concentrations (MICs) are indicated.

**Supplementary Figure 4: Phylogenetic tree of *pbp2x*.** The sequence clusters (ST344, ST448 and other), geographical location and minimal inhibitory concentrations (MICs) are indicated.

**Supplementary Figure 5: Phylogeography and sequence variation of ST448.** The maximum likelihood tree was constructed using substitutions outside of recombination events as described ([Croucher, et al. 2011](#_ENREF_2)). Shaded boxes indicate isolates with a penicillin MIC >0.06 µl/ml. Chromosomal locations of the putative, multiple (illustrated in red) and single (blue) recombination events detected in each terminal taxon are indicated by the top scale. Genome locations of some relevant genes conferring antibiotic resistance are also shown. ICE_1_*Sp*ST344 and ICE_2_*Sp*ST344 are specific Integrated Conjugative Elements (ICE). See text for details.

**Supplementary Figure 6: Phylogeography and sequence variation of ST344.** The maximum likelihood tree was constructed using substitutions outside of recombination events as described ([Croucher, et al. 2011](#_ENREF_2)). Shaded boxes indicate isolates with a penicillin MIC >0.06 µl/ml. Chromosomal locations of the putative, multiple (illustrated in red) and single (blue) recombination events detected in each terminal taxon are indicated by the top scale. Genome locations of some relevant genes conferring antibiotic resistance are also shown. ICE_1_*Sp*ST344 and ICE_2_*Sp*ST344 are specific Integrated Conjugative Elements (ICE). See text for details.

**Supplementary Figure 1**: Electron micrograph of the phage isolated from the nonencapsulated strain 110.58 (ST344).

**
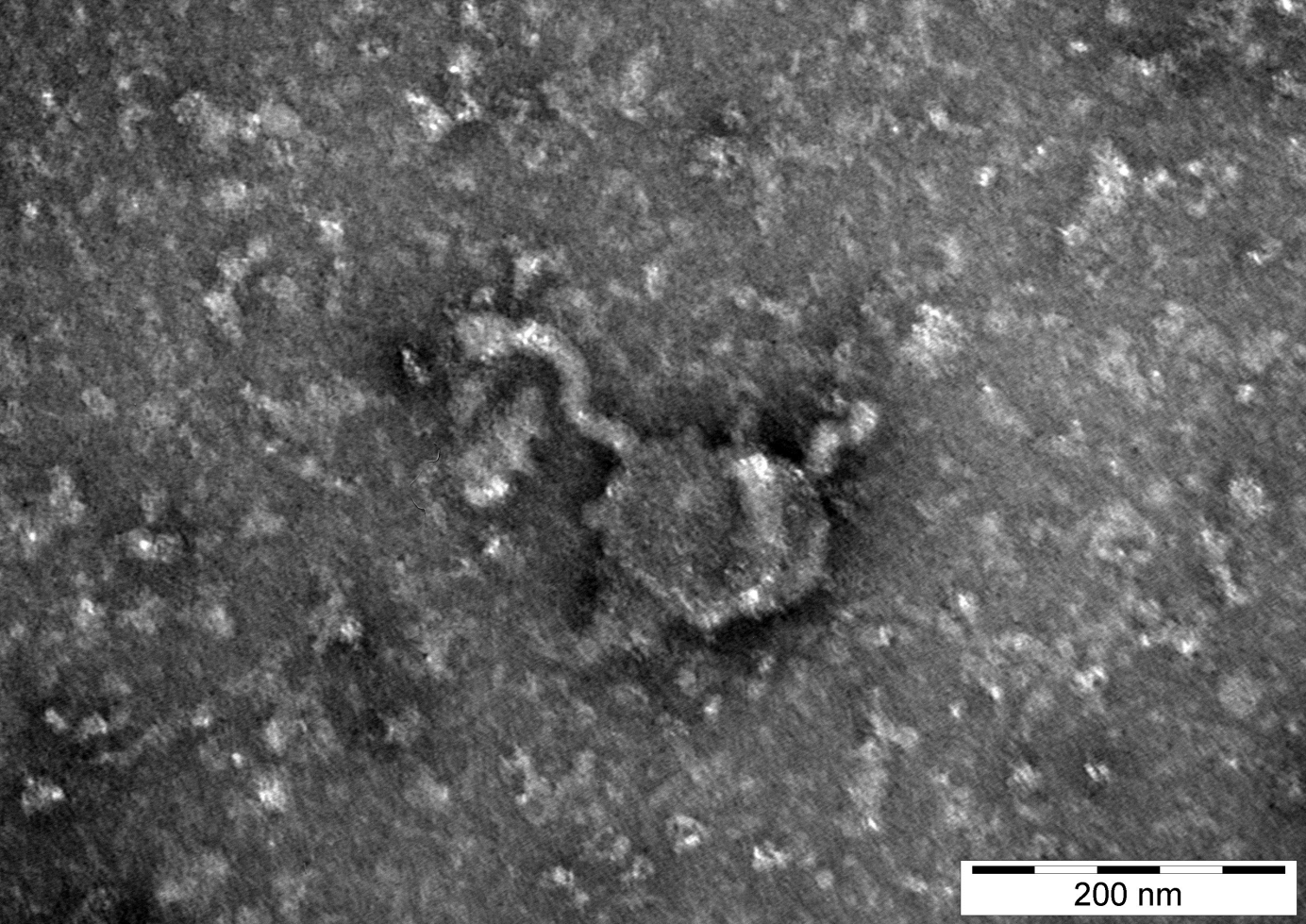
**

**Supplementary Figure 2: Phylogenetic tree of *pbp1a*.**


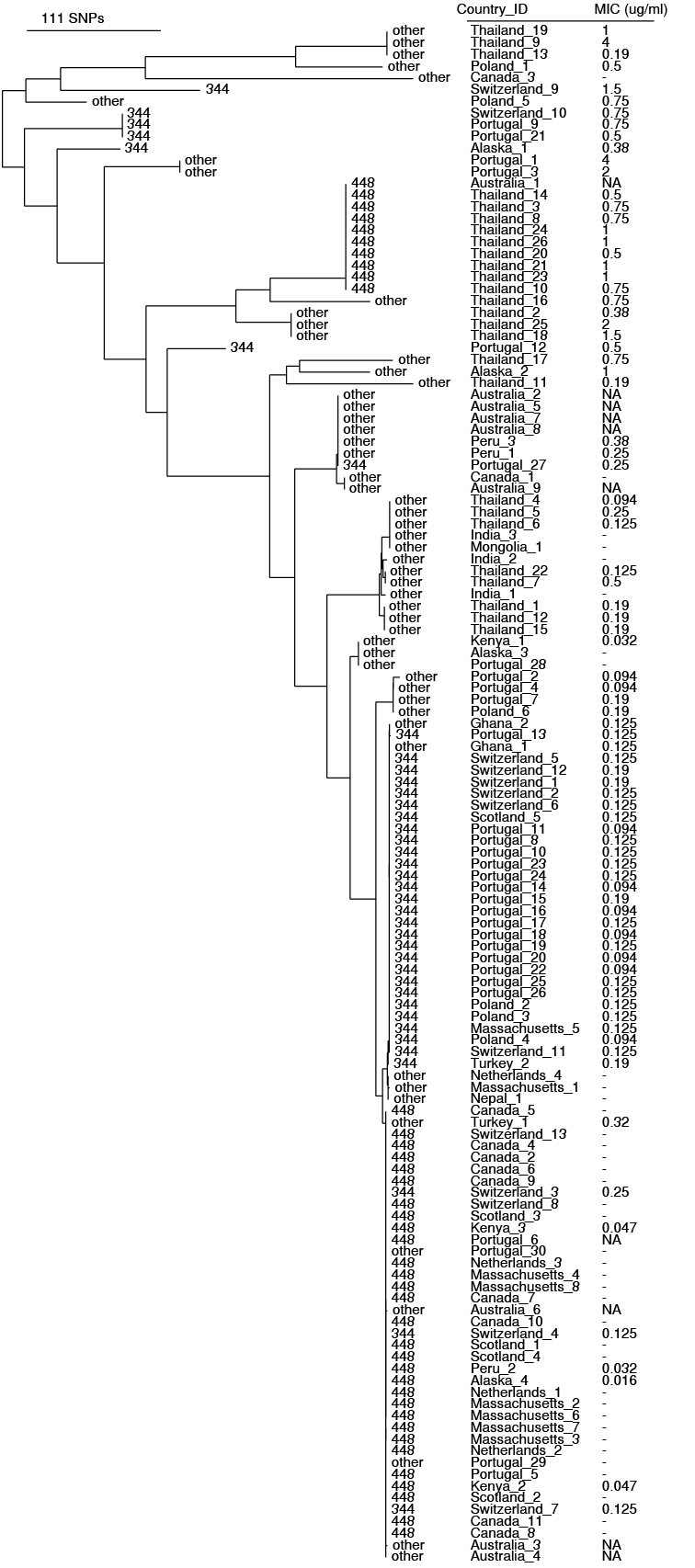


**Supplementary Figure 3: Phylogenetic tree of *pbp2b*.**


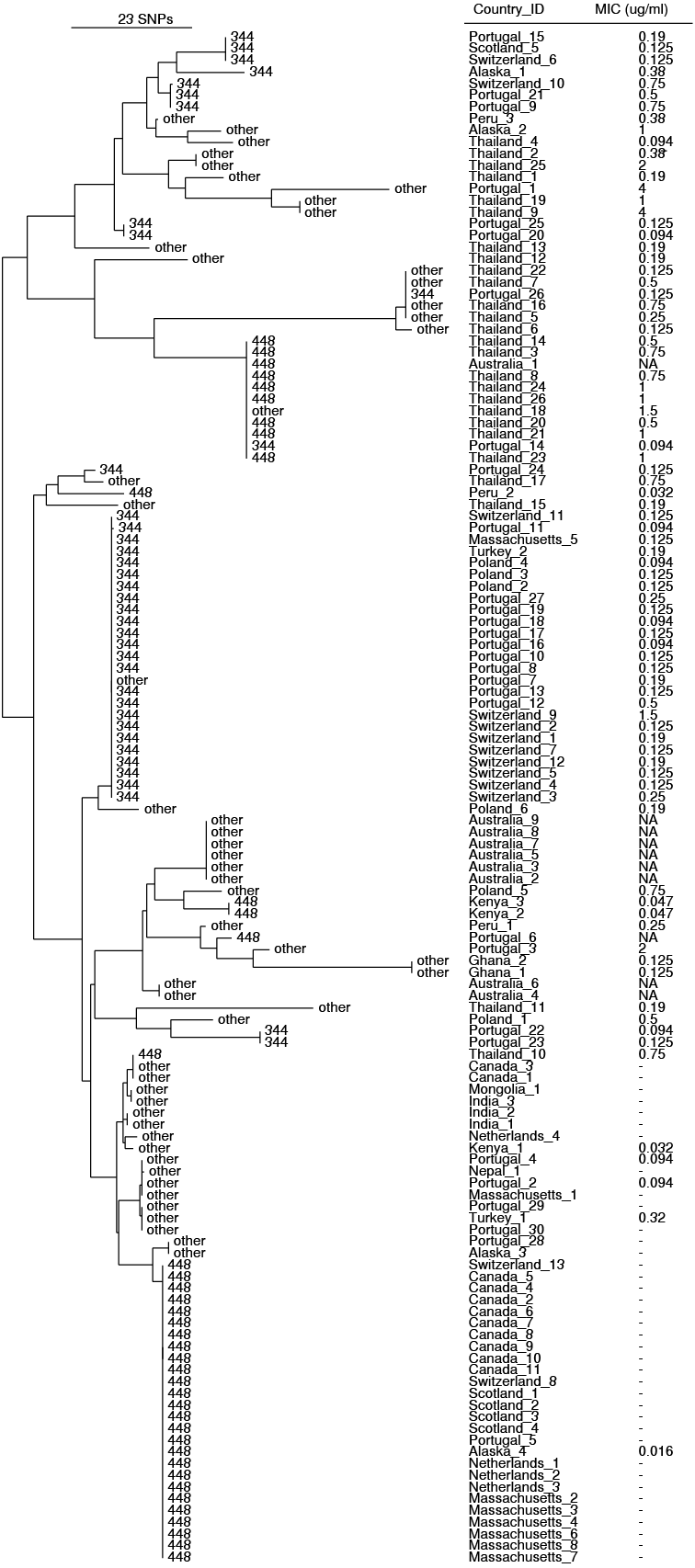


**Supplementary Figure 4: Phylogenetic trees of *pbp2x*.**


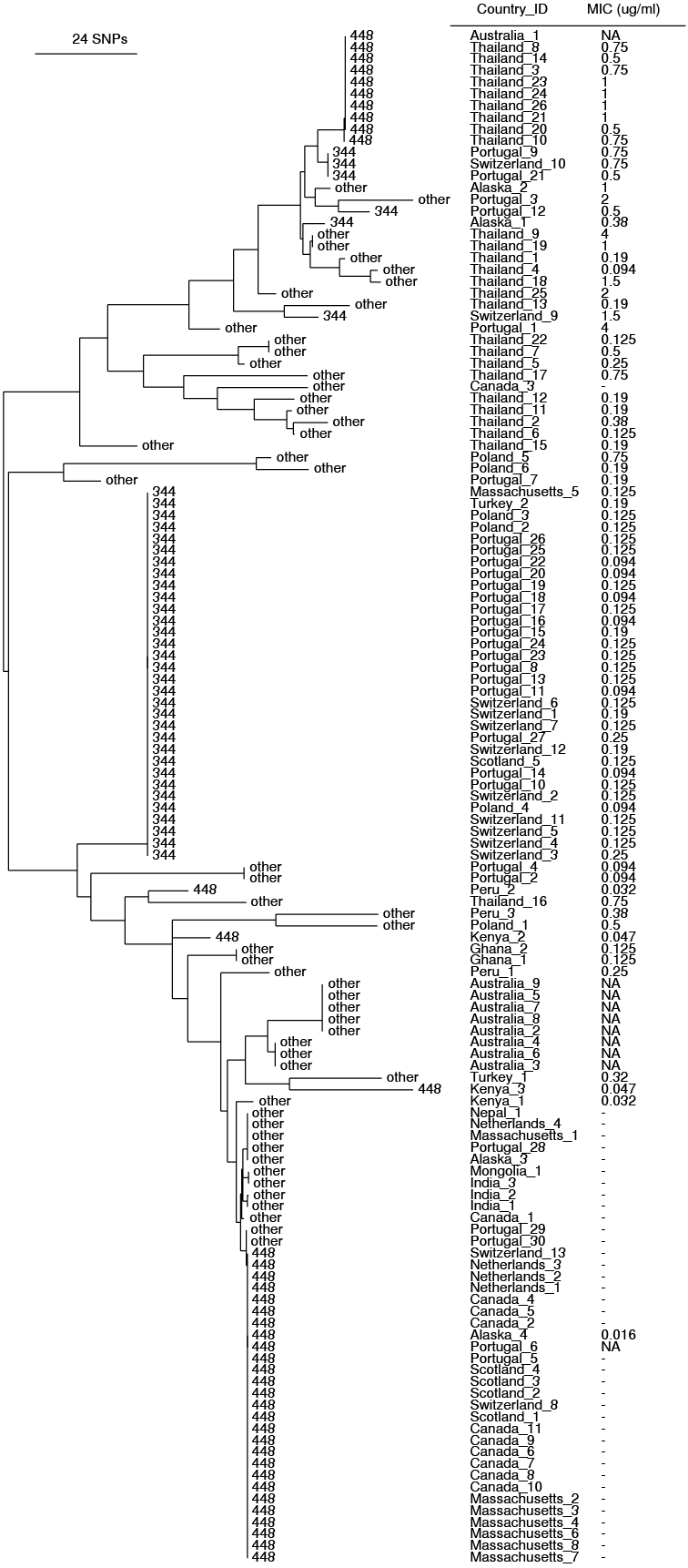


**Supplementary Figure 5: Phylogeography and sequence variation of ST448.**

**Supplementary Figure 6: Phylogeography and sequence variation of ST344.**
